# Supplementary material for: Association of cognitive reserve with 9-year domain-specific cognitive trajectories and risk of cognitive impairment in Mexican older adults
Source: PLoS One. 2025 Dec 10;20(12):e0338596. doi: 10.1371/journal.pone.0338596 (PMC12694827; doi:10.1371/journal.pone.0338596)
Supplement: Attachment — Submitted filename: Response to Reviewers.docx [file pone.0338596.s002.docx]

Dear Dr. Antoine Coutrot,

We hope this letter finds you well. We would like to express our sincere gratitude for the valuable feedback provided by the reviewers and for the time and effort dedicated to evaluating our work.

We are writing to formally answer the review comments in the following table.

| **Reviewer #1** | | |
| --- | --- | --- |
| **Review** | **Answer** | **Lines** |
| About operationalization of variables: Physical activity is treated as a dichotomous variable, which limits interpretability. I recommend highlighting this limitation more explicitly in the discussion. | We thank you for your valuable observation. We added a sentence highlighting this limitation in the discussion. | 403 - 406 |
| About Methods clarity: I consider that the choice of a -1.5 z-score cutoff for defining cognitive decline should be justified with additional references or explanation. | We included a sentence and some references to justify the parameters of cognitive impairment used. | 200 - 204 |
| About Discussion: Please expand the discussion on missing CR proxies (e.g., verbal intelligence, quality of education), which could influence the interpretation of your findings. | We incorporated arguments about the relevance of verbal intelligence as a possible reflection of the quality of education from a more dynamic perspective. | 429 - 431 |
| Since the average years of education in your sample are relatively low, it would be valuable to highlight the implications for populations with limited educational attainment and for public health interventions in low-resource settings. | We thank you for your valuable feedback, we added a paragraph highlighting our findings and its possible implications for public health interventions. | 420 - 424 |
| Ensure consistent use of terminology (“cognitive decline” vs. “cognitive impairment”) throughout the manuscript. | Additional to your recommendation, Reviewer #2 also pointed out the inconsistency throughout the manuscript and suggested the use of “cognitive impaired group”. We selected this concept because we cannot ensure that all participants experienced a process of decline, for instance, some fluctuated between normal cognition and the cognitively impaired group across the waves. Using the term “decline” would imply a progressive deterioration that we cannot confirm. Therefore, we replaced “decline” with “impairment” throughout the manuscript. | - |
| **Reviewer #2** | | |
| **Review** | **Answer** | **Line** |
| 1. In the description of the methods, especially when defining CR variables, I am having difficulty to understand how you deal with the occupational complexity for older adults. Do the retired participants keep their last occupation for data ? If authors did it that way, it may be difficult to justify this variable as dynamic component of CR. Moreover, does volunteer work, or partial-time employment have been recorded in MHAS data ? | To assess occupational complexity, we combined information from two databases of the same sample to address missing data. One database contained detailed job classifications, which were recoded as described in the Methods. The simplified version only specified whether participants were employed, unemployed, engaged in household chores, or seeking employment. Because this information varied across the waves, we considered the status reported for each wave. Participants who were unemployed were assigned the lowest score.  Regarding volunteering, participants reported this activity in a different section of the survey. We classified it as a leisure activity, consistent with the definition proposed by Elsden et al. (2022) as “voluntary, non-work, enjoyable activities engaged in during free time” (p. 1).  We specified these points directly in the Methods section (both in occupational complexity and leisure activities).  Partial-time employment is not considered in the MHAS, and in the context of working conditions in Mexico, full-time jobs usually consist in 48 hours per week, which can be extended by the time used to commute. | 150 – 155  162 - 164 |
| 2. In the logistic regressions investigating the risk of cognitive impairment, explained by CR variables, authors explained the creation of a composite numeric score, ranging from 0 to 95 points. If I read correctly that score, 60 points out of 95 are only calculated based on visual attention ? Why highlighting that cognitive skill more than others ? This method could focus too much on attentional skills and executive functions, influencing the findings of the association with dynamic CR | We appreciate your precise observation. As noted, 60 out of 95 points were related to visual attention. We decided to use these scores because they have been validated and standardized in other studies cited in the paper. However, we consider your observation to be a valid argument and is one of the reason why we chose to also perform separated statistical analyses for each cognitive domain.  We added a paragraph as a limitation of our study in the Discussion. | 406 - 409 |
| 3. Which were the 12 comorbidities authors adjusted for ? I understand that they attributed one point for each comorbidity for the total score, meaning that “hypertension” and “history of cancer” are treated the same ? I think that we should consider cardiovascular risk factors and Lancet’s 2024 risk factors of Dementia as serious cofounders in cohorts of older adults, even if they have few comorbidities like in the MHAS Study. | We already described the 12 comorbidities in the Methods section. These comorbidities were self-reported by participants and their caregivers, which represents a limitation of the MHAS. However, although self-reported, the questionnaire specifies that the condition must have been diagnosed by a doctor or medical personnel. We added a sentence regarding this specification in the Methods section.  We appreciate your suggestion, unfortunately, the MHAS does not provide that information, because it is a survey which started in 2001.  We agree that it is a limitation of the study and of the MHAS itself. We included a sentence in the Discussion addressing the relevance of considering specific comorbidities associated with the risk of dementia. | 208 -211  431 - 434 |
| 4. In abstract, last sentence indicates that increasing the years of education should delay cognitive decline, do authors really think there is fewer cognitive decline or do people start from higher CR and take more time to get MCI or dementia, whether they have a real cognitive decline ? | We thank you for your observation. We agree with the theoretical explanation described in the introduction of Stern (2012) that suggests that patients with Alzheimer’s Disease with higher CR can tolerate more pathology before manifesting clinical symptoms.  We changed the last sentence in the Abstract to address this view. | 36 - 37 |
| 5. In the end of the introduction, I would use the term “cognitive decline” only, instead of “MCI or dementia” because of the lack of data concerning functional living, or aetiologic of cognitive impairment. The expression “cognitive impaired group” line 206 is also convincing. | We appreciate your recommendation, which was also noted by Reviewer #1, regarding the inconsistency in terminology throughout the manuscript. Following your suggestion, we decided to use the term “cognitive impaired group”. We selected this term because we cannot ensure that all participants experienced a process of decline, for instance, some of them fluctuated between normal cognition and the cognitively impaired group across the waves. Using the term “decline” would imply a progressive deterioration that we cannot confirm. Therefore, we replaced “decline” with “impairment” throughout the manuscript. | - |
| 6. In the methods, authors should explain how occupational complexity, leisure activities, socioeconomic status were expressed numerically | In the Methods section we added quantitative information regarding occupational complexity, leisure activities, and socioeconomic status in their respective subsections. | - |
| 7. Authors explained their choice in discussion, but they were no possibility to precise the type and level of physical activity in your data ? | Unfortunately, the MHAS only has limited information about physical activity: if they took part in exercise or hard physical work three times a week or more.  We included a sentence in the Discussion section to detail about this limitation of the study. | 403 - 406 |
| 8. Do authors can justify the choice of -1.5SD to define cognitive impairment, instead of -2SD like majority of cognitive tests ? | We included a sentence and some references to justify the parameters to define cognitive impairment used. | 200 - 204 |
| Is there any patient that passed from cognitive impaired group to unimpaired across the waves, by changing of decade for example ? | We added a sentence in the Sociodemographic characteristic subsection of the Results to report the number and percentage of participants who reverted from the cognitively impaired group to the normal group.  Additionally, we included a paragraph in the Discussion to interpret these results and cited relevant studies to support our findings. | 242 – 247  394 - 399 |
| 9. Was there a visual or auditive evaluation before passing neuropsychological tests ? That may be relevant for visual attention. | A verification question regarding the use of lenses was asked prior to the neuropsychological assessments. If participants required lenses but did not have them, the assessment was not conducted.  This verification question was not applied for auditory tests. | - |
| 10. In Table 1, I would add a line to express the percentage of cognitively impaired participants for each wave. | We included a line in Table 1 with the percentage of cognitively impaired participants, and a brief description in the manuscript.  We also added a paragraph in the Discussion to analyze these findings as mentioned above. | 394 - 399 |
| 11. In discussion line 290, I agree with the differences made between physical activities and leisure activities, but it’s hard for me to understand the last sentence « related to a more comprehensive healthy aging ». Maybe authors should develop further their idea. | We expanded the sentence and supported it with a paper regarding leisure activities and their mechanism of action. | 319 - 321 |
| 12. Very interesting point to highlight that lower levels of education keep associated with cognition, these levels are rare in other studies focusing on higher degrees especially in WEIRD countries. Is it possible to try to categorise by diploma ? Primary, high school, college ? | We thank you for this valuable observation. However, we decided to use “years of education” rather than categorizing levels of education for several reasons: a) If a participant did not complete a given level, some years of education would not be accounted for, and b) categorization by diploma makes cross-national difficult due to differences in nomenclature and the ranges between levels. | - |
| 13. Is there much correlation between level of education and occupationnal complexity ? Maybe there is a circular effect on theses 2 variables | We did not conduct correlations beyond the primary aims of the study in order to avoid increasing the False Discovery Rate. However, we have included the correlation results in this letter for transparency. As you can observe, although statistically significant, the correlations are negative and of very weak magnitude. | - |

Kind regards,

Martina Ferrari Díaz
